# Production of constitutive and induced secondary metabolites is coordinated with growth and storage in Norway spruce saplings

**DOI:** 10.1093/treephys/tpaa040

**Published:** 2020-04-09

**Authors:** Jianbei Huang, Alexander Rücker, Axel Schmidt, Gerd Gleixner, Jonathan Gershenzon, Susan Trumbore, Henrik Hartmann

**Affiliations:** 1 Department of Biogeochemical Processes, Max Planck Institute for Biogeochemistry, Hans-Knöll-Str. 10, Jena 07745, Germany; 2 Department of Biochemistry, Max Planck Institute for Chemical Ecology, Hans-Knöll-Str. 8, Jena 07745, Germany

**Keywords:** carbon allocation, carbon limitation, growth, non-structural carbohydrate storage, phloem transport, secondary metabolites

## Abstract

A mechanistic understanding of how trees balance the trade-offs between growth, storage and defense is limited but crucial for predicting tree responses to abiotic and biotic stresses. Here we investigated how trees allocate storage of non-structural carbohydrates (NSC) to growth and constitutive and induced secondary metabolites (SM). We exposed Norway spruce (*Picea abies*) saplings to 5 weeks of complete darkness to induce light and/or carbon limitation and then applied methyl jasmonate (MeJA) to simulate biotic attack. We measured changes in biomass, NSC (sum of soluble sugars and starches), and constitutive and induced SM (sum of phenolic compounds and terpenoids) in current-year developing and previous-year mature needles and branches, as well as volatiles emitted from the canopy. Under darkness, NSC storage was preferentially used for constitutive biosynthesis of monoterpenes rather than biosynthesis of stilbenes and growth of developing organs, while SM stored in mature organs cannot be remobilized and recycled. Furthermore, MeJA-induced production of SM was constrained by low NSC availability in developing organs but not in mature organs grown in the dark. Emissions of volatiles were suppressed in the dark but after 1 h of re-illumination, emissions of both constitutive and induced monoterpene hydrocarbons recovered rapidly, whereas emissions of linalool and sesquiterpene produced via de novo synthesis did not recover. Our results highlight that light and/or carbon limitation may constrain constitutive and JA-induced biosynthesis of SM in coordination with growth, NSC storage and mobilization.

## Introduction

Increasing evidence suggests that trees are more susceptible to biotic attacks under abiotic stress (Seidl et al. 2017, Goodsman et al. 2018, Klein and Hartmann 2018). However, the underlying physiological mechanism is largely unknown. As sessile organisms, trees defend against biotic attacks through strategic allocation of resources to primary (e.g., growth and storage) and secondary metabolism (e.g., protection and defense). Understanding how trees balance trade-offs in resource allocation is thus of critical importance for understanding tree defense response under abiotic (e.g., drought and competition) and biotic stress (e.g., attack by insects and pathogenic microbes) (Huang et al. 2020).

Trees defend against insects and pathogens using a complex suite of biochemical mechanisms, including both constitutive secondary metabolites (SM) (e.g., always present) to avoid initial attack and induced SM to limit damage after attack. However, the biosynthesis of these metabolites requires considerable resources such as non-structural carbohydrate (NSC) substrates, which may incur a significant carbon cost (Züst and Agrawal 2017). As such, SM response to environmental stress has long been predicted to be driven by changes in carbon availability as a result of the balance between carbon supply via photosynthesis and carbon demand for growth, whereby SM decrease more than growth under carbon limitation (growth-differentiation balance hypothesis; Herms and Mattson 1992). Although a number of studies have investigated SM response to elevated CO_2_ and ozone in trees over the Past two decades, dynamics of SM have rarely been addressed in the context of environmental stress (severe drought, shade and defoliation) that reduces carbon supply (Holopainen et al. 2018).

Trees are known to store large amounts of NSC that can buffer the asynchrony of supply and demand on a timescale of months to decades (Dietze et al. 2014, Hartmann and Trumbore 2016). When trees are exposed to environmental stress, allocation to constitutive and induced SM may thus depend on the mobilization of NSC storage rather than newly fixed photosynthates (Najar et al. 2014, Sevanto and Dickman 2015). Trees should develop a balanced defense strategy by allocating storage proactively to growth and production of constitutive SM, while also retaining storage for induced SM when needed (Züst and Agrawal 2017). How such trade-offs have been shaped over evolutionary timescales and along environmental gradients has been debated intensely (Moreira et al. 2014, Pellissier et al. 2016, Galmán et al. 2018), while the phenotypic plasticity of such a trade-off strategy has not been given much consideration.

Assessing SM dynamics at the whole-tree level can be complicated by organ ontogeny. For example, young developing leaves usually have lower photosynthetic rates (van Dam et al. 1996) but a higher carbon demand for growth than older leaves. Under resource limitation, young developing leaves may thus rely on the mobilization of NSC storage for growth and production of constitutive SM (Huang et al. 2019). By contrast, in older organs, stored NSC and SM may act as carbon sources or might be used for other critical functions (e.g., osmoregulation and defense), which make them unavailable for export or catabolic processes (Martínez-Vilalta et al. 2016). For example, phenolic compounds are stored in phloem parenchyma cells at maturity senesce and become suberized, thereby imposing a physical and chemical barrier for recycling (Franceschi et al. 2005). Terpenoids in Pinaceae are stored in resin ducts where oleoresins accumulate under pressure in the extracellular lumen (Franceschi et al. 2005) and thus cannot be mobilized until insect or wounding damage occurs. In addition, young developing organs have often less NSC storage but produce more SM than mature organs after biotic attacks (Miller et al. 2005), and thus may be more affected by low resource availability. Hence, a comprehensive assessment of tree defense requires analysis of SM in organs of different ontogenetic stages.

Trees respond to abiotic and biotic stress not only by increasing concentrations of SM that are stored in tissues but also by emitting a blend of volatiles (Loreto and Schnitzler 2010). These volatiles play a variety of roles, ranging from protection against oxidative stress by scavenging reactive oxygen species (ROS) to defense by repelling and poisoning insects (Raffa 2014) or attracting the herbivores’ natural enemies (McCormick et al. 2012). In Pinaceae, large proportions of terpenoid volatiles are likely emitted from resins, but there is also evidence that terpenoid volatiles are synthesized de novo (Ghirardo et al. 2010), even under reduced carbon supply (Huang et al. 2018). However, studies on how carbon availability may alter induced emissions of terpenoids, particularly monoterpenes and sesquiterpenes are still sparse.

Norway spruce (*Picea abies*) is a dominant tree species in Europe. Due to the ongoing climate change, spruce forests are experiencing more frequent and more severe biotic attacks by insects such as spruce sawfly, aphids, bark beetles and budworms (Newton 2007, Seidl et al. 2016, Biedermann et al. 2019). Reduced carbon supply during climate change-related stress events like drought or heat spells has been proposed to compromise tree defense against biotic attacks (McDowell et al. 2011). However, our previous work showed that spruce saplings exposed to low carbon supply invest carbon into phenolic compounds and monoterpenes at the cost of storage and growth (Huang et al. 2019). Canopy emissions of monoterpenes and sesquiterpenes from spruce saplings are sustained and partly fueled by newly assimilated carbon even under a negative carbon balance (50 p.p.m. [CO_2_]) (Huang et al. 2018). However, these studies did not address trade-offs with induced SM. Jasmonate signaling has been recognized as an important mechanism through which plant respond to wounding and herbivory (Howe 2004, Züst and Agrawal 2017). Early studies in Norway spruce demonstrated that applications of methyl jasmonate (MeJA) triggered accumulation of terpenes and phenolics in both saplings grown in controlled conditions (Martin et al. 2002, 2003) and mature trees grown in the field (Erbilgin et al. 2006, Schiebe et al. 2012).

To fill the knowledge gap, we performed an experiment that was specifically designed to induce light and carbon limitation and simulate insect attack. Spruce trees were exposed to 5 weeks of complete darkness, a common approach to simulate carbon limitation (Sevanto et al. 2014, Piper and Fajardo 2016, Wiley et al. 2017, Weber et al. 2018), followed by spraying of MeJA to trigger plant defense responses to insect attack (Martin et al. 2002, Hudgins et al. 2003, Miller et al. 2005). We measured changes in biomass growth of current-year, developing branches and analyzed primary (soluble sugars and starch) and secondary (flavan-3-ols, stilbenes and terpenoids) metabolites stored in different organs (current-year developing and previous-year mature needles and branches), as well as volatile compounds emitted from the canopy. Our main objective was to elucidate whether and to what extent constitutive and induced SM are sustained by carbon storage and occur at the cost of growth. We specifically hypothesized that (i) NSC storage is preferentially used for growth rather than for producing constitutive SM in developing, sink organs, while constitutive SM remained relatively constant in older, source organs; (ii) spraying MeJA depletes NSC storage and induces the biosynthesis of SM, but the depletion and induction by MeJA are greater when plants are grown in the light than in the dark. Furthermore, we expect that (iii) the amount of MeJA-induced volatile emissions is constrained by light and/or carbon availability.

## Materials and methods

### Plant material

Young Norway spruce clones (*Picea abies*, S21K0420117) were purchased from the Skogforsk tree improvement agency in Sweden. Clones were propagated from cuttings of a 3-year-old Norway spruce in 2004. Clones were originally grown in natural soil but then transplanted into pots filled with sand and amended with a slow-releasing inorganic fertilizer (Osmocote Start, Everris International BV, Geldermalsen, The Netherlands). All saplings were grown in an outdoor area at Max Planck Institute for Biogeochemistry (MPI-BGC, 50° 54′ 36.24″ N 11° 33′ 59.95″ E) since 2014.

### Growth chambers, treatments and samplings

**Figure 1.**
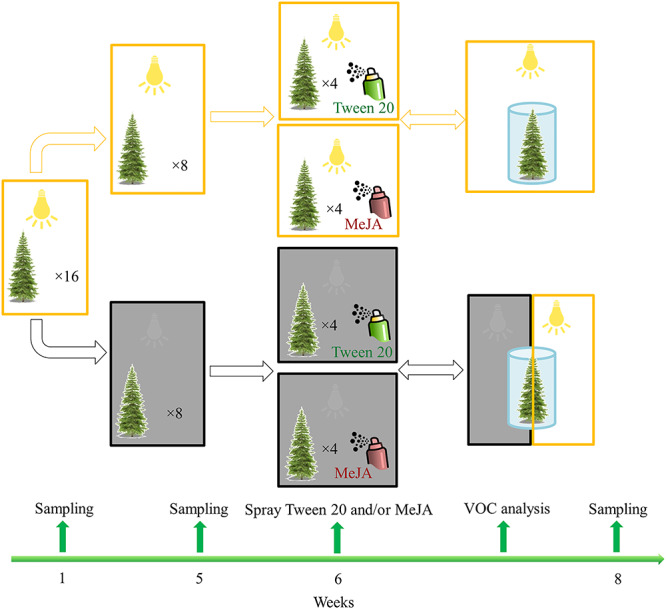
Schematic of the treatments and the timeline. A total of 16 saplings were transferred to phytochambers and acclimated for 1 week (week 1). Half of the saplings (*n* = 8) were then exposed to complete darkness for 5 weeks, while the other half were kept in the light, to generate contrasting situations of light and carbon availability. Saplings were then sprayed with either MeJA dissolved in Tween 20 or Tween 20 only at week 6, yielding in total four treatments (*n* = 4). Sampling was carried out prior to darkening (week 1), after the darkness treatment (week 5) and after the MeJA treatment (week 8). Methyl jasmonate was sprayed 5 days after sampling at week 5. Volatiles were measured between week 6 and week 7. Note that trees grown in the dark treatment were measured in the dark before re-exposing them to light and were then measured again VOC, volatile organic compounds.

In May 2017, 16 clones at similar size (1.6–1.8 m tall with basal stem diameter between 2.6 and 2.9 cm) were transferred and randomly assigned into phytochambers (York Refrigeration, Mannheim, Germany), and acclimated for 1 week to the following conditions: light/dark, 16/8 h; photosynthetic photon flux density, *c.* 800 μmol m^−2^ s^−1^; temperature, 25 °C; relative humidity, 60%. After this period, saplings were exposed to factorial treatments that induced light and carbon limitation via darkness, followed by a spray application of MeJA to induce defenses (Figure 1). We exposed half of the saplings to complete darkness while keeping the other half under the original light/dark regime, to generate contrasting situations of carbon availability. After 5 weeks of darkness, we sprayed 10 mM MeJA (Sigma–Aldrich, USA) in 0.1% (v/v) Tween 20/water on half of the saplings grown in the dark and on half of the saplings under the light/dark regime; the remaining saplings were sprayed with 0.1% Tween 20 alone as a control. Methyl jasmonate solution was sprayed onto the whole canopy with a spray gun (1 l per sapling). Overall, 16 saplings were divided into four treatments (*n* = 4): (i) spraying Tween 20 (light-Tween) on trees grown under the light/dark regime; (ii) spraying MeJA (light-MeJA) on trees grown under the light/dark regime; (iii) spraying Tween 20 (dark-Tween) on trees grown for 5 weeks in the dark; and (iv) spraying MeJA (dark-MeJA) on trees grown for 5 weeks in the dark (Figure 1). The different treatments were carried out in separate phytochambers to avoid cross-contamination of MeJA.

The experiment started just after bud break. For each sampling, we collected developing, non-lignified shoots (needles and branches) grown during the experiment in the current year and shoots grown in the previous year (referred to as developing and mature, respectively). Approximately 10–15 fresh shoots (*c.* 15–20 g) were collected randomly using a sharp branch cutter. Developing shoots were separated immediately from the mature shoots using a scissor. Both developing and mature shoots were flash-frozen in liquid nitrogen and then transferred to a −80 °C freezer for later processing. Sampling was carried out prior to darkness (week 1), after the darkness treatment (week 5), and after spraying Tween 20 or the mixture of Tween and MeJA (week 8) (Figure 1). To reduce potential effects from daytime variations in carbon metabolism (Smith and Stitt 2007), sampling was always conducted between 14:00 and 17:00 h. To avoid releases of large emissions of terpenoids in response to sampling wounding, MeJA was sprayed 5 days later after sampling at week 5. Our previous work showed that 4 days are sufficient for wound healing (Huang et al. 2018), while terpenoid accumulation in tissues of spruce saplings is achieved 2 weeks after MeJA treatment (Martin et al. 2002). At week 8, we destructively harvested the plants to determine the dry biomass of the whole canopy.

### Biomass processing

Prior to metabolite analysis, needles and branches were separated, homogenized while immersed in liquid nitrogen in a mortar and then split into two parts. For analysis of terpenoids, part of the sample was ground in liquid nitrogen using mortar and pestle and then stored at −80 °C. For analysis of soluble sugars and phenolic compounds, the other part of the sample was weighed fresh, transferred into pre-cooled wide-neck filter flasks (Martin Christ, Osterode, Germany) and attached to a freeze dryer (Dieter Piatkowski, Munich, Germany) for 48 h. The freeze-dried samples were then weighed again to determine fresh-to-dry weight conversion factors. Freeze-dried samples were then ground to fine powder in a ball mill (Retsch® MM400, Haan, Germany) and stored at −20 °C.

### Soluble sugar and starch analysis

Soluble sugars and starch were extracted following the standard protocol developed by Landhausser et al. (2018). Briefly, ~10 mg of soluble sugars were extracted with 0.5 ml of 85% ethanol, vortexed for 1 min, incubated at 90 °C for 10 min and centrifuged at 13,000*g* for 1 min. The supernatant was collected and the pellet was re-extracted twice using the same procedures. Supernatants were combined and then diluted and analyzed with a High-Performance Liquid Chromatography coupled to a Pulsed Amperometric Detection (HPLC-PAD) following the protocol of Raessler et al. (2010). The concentrations of glucose, sucrose and fructose were summed and are reported here as total soluble sugars. Starch from the remaining pellet was digested with 1.0 ml of α-amylase, vortexed for 1 min, incubated at 85 °C for 30 min and then centrifuged at 13,000*g* for 1 min. An aliquot of supernatants was collected and digested with amyloglucosidase (Sigma–Aldrich), incubated at 55 °C for 30 min following Landhausser et al. (2018). The glucose hydrolysate was then collected and measured by HPLC-PAD. Starch was calculated as glucose equivalents by multiplying by a factor 0.9.

**Table 1:** Fresh and dry average biomass (mg) of developing branches of *Picea abies* grown in the light or darkness (week 5), followed by spraying MeJA dissolved in Tween 20 (L-JA, D-JA) or Tween 20 only (L-TW, D-TW) at week 6 and harvested at week 8. Values are means ±1 SD (mg). Student’s *t*-test and Tukey’s honest significance test (*P* < 0.05) were used to test the differences between means at week 5 (*n* = 8) and week 8 (*n* = 4) (*P* < 0.01), respectively. Different letters indicate significant differences between different treatments.

Week 5			Week 8		
Treatment	Fresh	Dry	Treatment	Fresh	Dry
Light	66.1 (22.6) a	25.6 (8.7) a	L-TW	80.6 (16.8) a	35.8 (8.9) a
			L-JA	71.7 (22.7) a	33.2 (9.4) a
Dark	33.6 (8.5) b	9.0 (2.3) b	D-TW	31.1 (10.6) b	10.6 (3.5) b
			D-JA	29.3 (3.1) b	12.5 (1.5) b

### Phenolic compound analysis

Phenolic compounds were extracted as described in Huang et al. (2017), with slight modifications. Briefly, ~30 mg of ground freeze-dried sample was extracted with 1 ml methanol containing 20 μg of apigenin-7-glucoside (Carl Roth GmbH, Germany) as an internal standard. The mixture was vortexed for 10 min, bead-beaten for 40 s at 6.0 m s^−1^ (MP Biomedicals, Santa Ana, CA, USA) and centrifuged for 10 min at 13,000*g*. The supernatant was collected, and the pellet was re-extracted with 0.5 ml methanol containing the internal standard. Both supernatants were combined and analyzed using HPLC-mass spectrometry (MS) (HPLC, Agilent, Santa Clara, CA, USA; MS, Sciex, Darmstadt, Germany). Phenolic compounds were separated on a Zorbax Eclipse XDB-C18 column (4.6 x 50 mm, 1.8 μm; Agilent) using mobile phase 0.05% (v/v) formic acid (phase A) and acetonitrile (phase B) at a flow rate 1.1 ml min^−1^, with the following profile: 0–1 min, 100% A, 0% B; 1–7 min, 0–65% B; 7–7.01 min, 65–100% B; 7.01–8 min, 100% B; 8–8.01 min, 100–0% B; 8.01–10 min, 0% B. The MS was operated as follows: negative ionization mode; ion spray voltage, −4200 V; turbo gas temperature, 700 °C; nebulizing gas, 70 p.s.i.; curtain gas, 30 p.s.i.; heating gas, 60 p.s.i.; and collision gas at 10 p.s.i. Multiple reaction monitoring was used to analyze the parent ion → product ion: *m*/*z* 288.9 → 109.1 for catechin; *m*/*z* 304.9 → 125 for gallocatechin; *m*/*z* 576.9 → 289.1 for proanthocyanidin B1; *m*/*z* 389 → 227 for piceid; *m*/*z* 404.8 → 243 for astringin; and *m*/*z* 418.9 → 257.1 for isorhapontin. The sum of catechin, gallocatechin and proanthocyanidin B1 was reported as flavan-3-ols. The sum of piceid, astringin and isorhapontin was reported as stilbenes. Compounds were identified by comparison of retention time and mass spectra with standards and quantified using the peak area in relation to the internal standard peak area. The response factors were calculated with standards (Huang et al. 2019). Linearity of quantification was confirmed by analyzing a gradient of catechin.

### Monoterpene analysis

Approximately 100 mg of ground fresh sample was extracted with 1 ml tert-butyl methyl ether (TBME) containing 30 μg ml^−1^ 1,9-decadiene (Sigma–Aldrich) as an internal standard. The extraction procedures used for phenolic compounds were also used for monoterpenes with slight modifications, and the supernatant was dehydrated using anhydrous MgSO_4_ before analysis. Compound separation was performed on an Agilent 6890 Series Gas Chromatograph (GC) (Santa Clara, CA, USA) with a DB5MS column (30 m × 0.25 mm × 0.25 μm) and carrier gas at a flow rate of 1.5 ml min^−1^. One microliter of sample was injected in splitless mode. The oven was programmed from an initial temperature of 40 °C (3 min hold), followed by an increase to 80 °C at 2 °C min^−1^ and to 180 °C at 5 °C min^−1^, and then heated to 300 °C for 2 min. The same GC was coupled to either an Agilent 5973N Mass Spectrometer for identification (carrier gas, helium; interface temperature, 280 °C; electron energy, 70 eV; source temperature, 230 °C) or a Flame Ionization Detector for quantification (carrier gas, hydrogen; operated at 300 °C). α-Pinene, camphene, β-pinene, myrcene, 3-carene, limonene and 1, 8-cineole were identified by comparison of mass spectra with that of the authentic standards and/or the reference spectra of databases (see Figure S1 available as Supplementary Data at *Tree Physiology* Online; Wiley 275, NIST 98, Adams 2205) and quantified with relative response factors (see Huang et al. 2019) estimated based on the effective C number concept (Scanlon and Willis 1985).

### Canopy volatile collection, identification and quantification

Canopy volatiles were collected and analyzed after spraying MeJA at week 6 (Figure 1). Trees were transferred to a different phytochamber, where the main canopy was enclosed in a cylindrical chamber (diameter, 70 cm; height, *c.* 50–70 cm) covered with fluorinated ethylene propylene (FEP) foil (see Huang et al. 2018 for more details). Chambers were flushed with *c.* 18 l min^−1^ volatile-free synthetic air (Westfahlen AG, Germany) premixed with CO_2_ at *c.* 400 p.p.m. Because of the potential confounding effects of light and carbon availability on volatile emissions, trees grown under dark treatments were initially measured in the dark, then re-exposed to light and measured again (Figure 1). We further tested whether volatile emissions were affected when saplings were re-exposed to light and synthetic air without CO_2_, in order to gain potential insights on the contribution of newly assimilated carbon during re-illumination and carbon storage. Note that each tree was sampled once under each of three conditions. Because stomatal closure in the dark may result in high intercellular volatile partial pressure in leaves (Niinemets and Reichstein 2003), volatiles were collected only 1 h after re-illumination to avoid potential volatile pulses after opening of stomata in response to re-illumination (Harley et al. 2014). Given the time required for preparation, tree acclimation and measurements, we could collect and measure volatiles from only one or two trees per treatment per day. Note that trees from different treatments were transferred and measured at different time and placed back directly after volatile collection.

**Figure 2.**
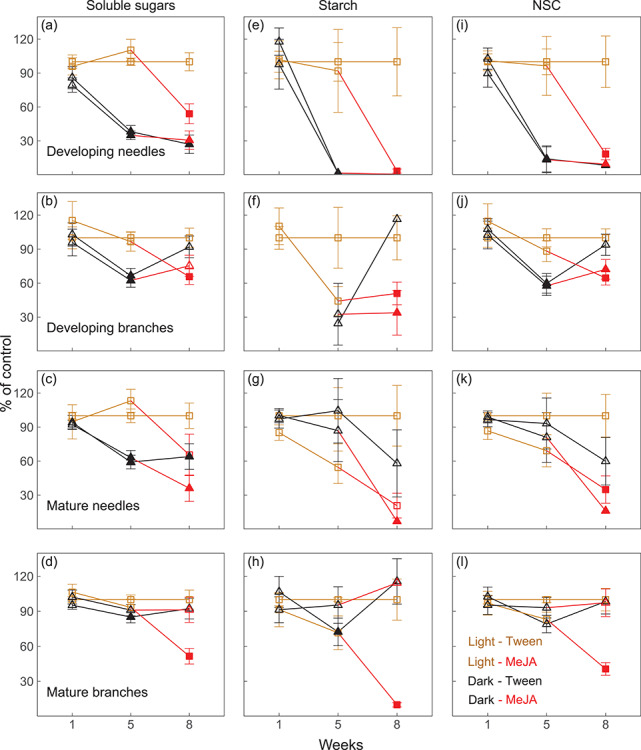
Percentage deviation of concentrations of soluble sugars (a–d), starch (e–h) and total NSC (soluble sugars + starch, i–l) in developing and mature needles and branches of *Picea abies* grown under light-MeJA (squares, yellow–red line), dark-Tween (triangles, dark line) and dark-MeJA (triangles, dark–red line), compared with control, light-Tween (squares, yellow line). The red lines indicate MeJA treatment under either light or darkness. Error bars indicate coefficients of variation and propagated standard errors. Significant differences between the treatments (light-MeJA, dark-MeJA, dark-Tween) and control (light-Tween) were calculated based on the raw concentrations and are indicated by filled symbols (*P* < 0.05).

We used a mobile GC-MS, HAPSITE (Inficon, Switzerland), to identify and quantify volatiles emitted from the canopy enclosed in FEP foil (Huang et al. 2018). Volatiles were collected using a tri-bed concentrator filled with carbon-based absorbent at a flow rate of 100 ml min^−1^ for 5 min. The start-up temperatures were as follows: column, 60 °C; membrane, 80 °C; valve oven, 70 °C; heated lines, 70 °C; probe, 40 °C. Compound separation was performed on a 15 m fused silica Restek Rtx-1 MS capillary GC column (5% diphenyl/95% dimethyl polysiloxane phase, 0.25 mm inner diameter, 1 μm film thickness). Volatiles were desorbed from the concentrator using the following temperature program: 60 °C hold for 1 min, followed by an increase to 120 at 30 °C min^−1^, hold for 15 min, and then to 200 °C at 30 °C min^−1^, hold for 2 min. The MS was performed in the electron ionization mode at 70 eV with N_2_ as carrier gas. We identified monoterpenes including α-pinene, camphene, β-pinene, 3-carene, limonene and linalool and sesquiterpenes including β-farnesene, α-farnesene, α-bisabolene and methyl salicylate (MeSA) by comparing their mass spectra with the authentic standards and/or the reference spectra of NIST library. Monoterpenes and sesquiterpenes were quantified using the calibration curve of α-pinene. Volatile emission rates were calculated based on the dry mass of the canopy enclosed in the chamber.

### Data analysis

Shapiro–Wilk and Levene tests were used to check the normality and homogeneity of variances, respectively. Data were analyzed with a two-way ANOVA using darkness, MeJA and their interaction as independent variables. The aligned rank transform was applied when normality or homogeneity assumption were not met (Wobbrock et al. 2011). Depending on normality and homogeneity, the significance of differences in biomass of developing branches, in emissions of volatiles, were assessed with Tukey’s honestly significant difference test (HST) or Wilcoxon’s rank-sum test, and the significance of differences in concentrations of metabolites between the control (light-Tween) and each of the treatments (light-MeJA, dark-Tween, dark-MeJA) were determined with Student’s *t*-test or Wilcoxon’s rank-sum test. Concentrations of metabolites were expressed as the percentage of control within each sampling time point, the coefficient of variation served as the measure of spread in the control treatment and errors were propagated for other treatments.

We used multiple linear regressions to determine the relationships of NSC storage (i.e., source) to different SM in mature organs and to NSC in developing organs (sinks). For developing organs, we considered correlations of NSC (substrate) to SM and biomass growth. We also determined the relationship of monoterpene emissions to monoterpene concentrations in developing and mature organs. All statistical analyses were conducted in R (version 3.23, R Development Core Team 2016).

## Results

### Effects of darkening and MeJA on biomass growth of developing branches

Fresh and dry biomass of developing branches were significantly lower when saplings were grown in the dark than when in the light (*P* < 0.05, HST or Wilcoxon’s rank-sum test; Table 1). Application of MeJA at week 6 did not affect biomass growth of developing branches at week 8, whether in the light or in the dark. After 2 weeks of MeJA treatment, all developing needles withered and began to fall in the dark, but this reaction was much less severe in the light. By contrast, trees sprayed with Tween 20 alone did not show any symptoms of wilting response.

### Effects of darkening and MeJA on soluble sugars and starch

Trees in the control treatment (light-Tween) exhibited little variability in the concentrations of soluble sugars across organs. After darkness, the concentrations of soluble sugars were significantly reduced by *c.* 60% in developing needles and *c.* 40% in developing branches and mature needles, but only by *c.* 10% in mature branches, compared with the light treatment (*P* < 0.05, Student’s *t*-test or Wilcoxon’s rank-sum test; Figure 2a–d). In developing needles and branches, concentrations of starch decreased to almost zero and thus caused a strong decrease in total NSC (sugars + starch) relative to the control (Figure 2e, f, i and j). In mature organs, however, starch levels showed large within-treatment variability, and there were no significant differences in NSC concentrations across treatments (Figure 2g, h, k and l). Note that the concentrations of starch and NSC declined over time, compared with initial concentrations at week 1 (see Figure S2 available as Supplementary Data at *Tree Physiology* Online), likely due to large carbon demand for growth flush.

After spraying Tween 20 and MeJA at week 6, the concentrations of soluble sugars, starch and NSC significantly decreased in developing organs grown in the light compared with spraying Tween 20 alone (*P* < 0.05, Student’s *t*-test; Figure 2). The initial reductions of NSC from darkening did not continue during the experiment, and concentrations simply remained at low levels (~15–25 mg g^−1^), irrespective of MeJA treatment (Figure 2i and j). From the two-way ANOVA, we found significant interactions of darkness and MeJA on concentrations of soluble sugars, starch and NSC in developing needles (*P* < 0.01), but not in developing branches (*P* > 0.05; Table 2). Spraying MeJA further decreased concentrations of NSC in mature needles and branches grown in the light, as well as in mature needles grown in the dark, but not in branches (Figure 2c, d, g and h), where concentrations of soluble sugars and starch remained above 30 and 15 mg g^−1^, respectively, similar to control levels (see Figure S2 available as Supplementary Data at *Tree Physiology* Online). For concentrations of glucose, sucrose and fructose, please see Table S2 available as Supplementary Data at *Tree Physiology* Online. Note that during the experiment concentrations of NSC generally decreased in mature organs in all treatments (see Figure S2 available as Supplementary Data at *Tree Physiology* Online).

**Table 2:** Two-way ANOVA results for the effects of darkness treatment and MeJA and their interactions on the concentrations of NSC, flavan-3-ols, stilbenes and monoterpenes in developing needles and branches of *Picea abies*, as well as on volatiles from the canopy after re-exposure to light.

	Developing needles			Developing branches			Canopy volatiles		
Pools	Dark	MeJA	Dark × MeJA	Dark	MeJA	Dark × MeJA	Dark	MeJA	Dark × MeJA
Soluble sugars	<0.01	<0.01	<0.01	0.88	<0.01	0.13	–	–	–
Starch	<0.01	<0.01	<0.01	0.93	<0.01	0.21	–	–	–
NSC	<0.01	<0.01	<0.01	0.89	<0.01	0.22	–	–	–
Flavan-3-ols	<0.01	<0.01	<0.01	0.61	<0.01	0.81	–	–	–
Stilbenes	<0.01	0.20	0.29	<0.01	0.70	0.72	–	–	–
Monoterpenes	0.18	0.12	0.45	0.12	<0.01	0.10	0.10	<0.01	0.24
Linalool	–	–	–	–	–	–	<0.01	<0.01	<0.01
Sesquiterpenes	–	–	–	–	–	–	<0.01	0.27	0.32
MeSA	–	–	–	–	–	–	<0.01	<0.01	<0.01

For effects on individual soluble sugars and SM, please see Table S1 available as Supplementary Data at *Tree Physiology* Online.

### Effects of darkening and MeJA on flavan-3-ols, stilbenes and monoterpenes

**Figure 3.**
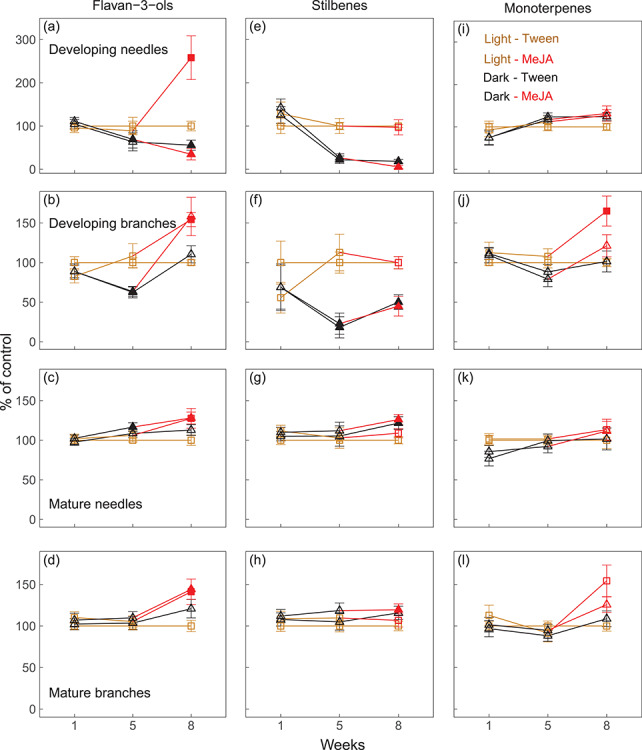
Percentage deviation of concentrations of flavan-3-ols (a–d), stilbenes (e–h) and monoterpenes (i–l) in developing and mature needles and branches of *Picea abies* grown under light-MeJA (squares, yellow–red line), dark-Tween (triangles, dark line) and dark-MeJA (triangles, dark–red line), compared with control, light-Tween (squares, yellow line). The red lines indicate MeJA treatment under either light or darkness. Error bars indicate coefficients of variation and propagated standard errors. Significant differences between the treatments (light-MeJA, dark-MeJA, dark-Tween) and control (light-Tween) were calculated based on the raw concentrations and are indicated by filled symbols (*P* < 0.05).

**Figure 4.**
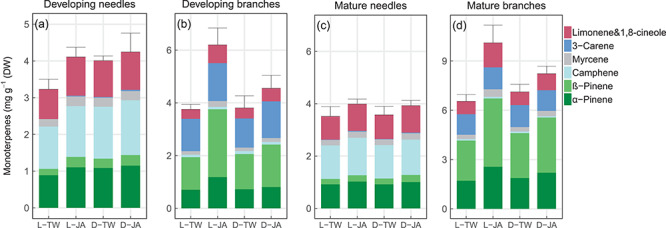
Concentrations (mg g^−1^ DW) of different monoterpenes in developing (a, b) and mature (c, d) needles and branches of *Picea abies* grown either in light or dark, followed by spraying MeJA, dissolved in Tween 20 or Tween 20 only: light-Tween (L-TW), light-MeJA (L-JA), dark-Tween (D-TW) and dark-MeJA (D-JA). Values are the means of four individual chambers; error bars represent ±1 SE (mg g^−1^ DW).

Trees in the control treatment (light-Tween) exhibited little variability in the concentrations of flavan-3-ols, stilbenes and monoterpenes across organs. Concentrations of flavan-3-ols and stilbenes were *c.* 40 and 80% lower in developing organs when grown in the dark than in the light, respectively (Figure 3a, b, e and f; *P* < 0.05, Student’s *t*-test); by contrast, neither total concentrations (Figure 3i and j) nor concentrations of individual monoterpenes (Figure 4a and b) were significantly affected by darkness (*P* > 0.05). In mature needles and branches, concentrations of both phenolic compounds and monoterpenes remained relatively constant or even slightly increased during darkness, compared with organs grown under light.

After spraying MeJA at week 6, concentrations of flavan-3-ols significantly increased across organs grown in the light and to a greater extent in developing than in mature organs (*P* < 0.05, Student’s *t*-test; Figure 3a–d). Concentrations of flavan-3-ols also increased in developing branches grown in the dark, in mature needles and branches even to levels similar to trees grown in the light (Figure 3b–d). However, contrasting patterns were observed in developing needles, where flavan-3-ols significantly increased by more than 150% when saplings were grown in the light but continued to decrease in the dark (Figure 3a). The differential effects resulted in significant interactions of darkness and MeJA on flavan-3-ols (*P* < 0.01; Table 2). Unlike flavan-3-ols, concentrations of stilbenes did not respond to MeJA in any of the organs tested (*P* > 0.05; Table 2, Figure 3e–h). Spraying MeJA increased concentrations of monoterpenes, but the effect was only significant for developing branches (Figure 3i–l), where α- and β-pinene and limonene increased more than camphene, myrcene and 3-carene (Figure 4b). Note that the concentrations of flavan-3-ols, stilbenes and monoterpenes were similar in developing needles and branches grown in the light (see Figure S3 available as Supplementary Data at *Tree Physiology* Online).

### Effects of darkening and re-illumination and MeJA on volatile emissions

**Figure 5.**
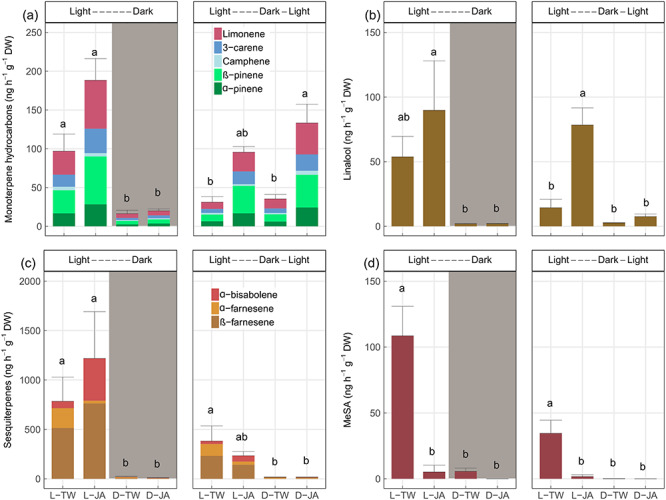
Emissions (ng h^−1^ g^−1^ DW) of volatiles, including monoterpene hydrocarbons (a), linalool (b), sesquiterpenes (c) and methyl salicylate (MeSA), (d) from the canopy of *Picea abies* grown either in light or dark, followed by spraying MeJA dissolved in Tween 20 or Tween 20 only: light-Tween (L-TW), light-MeJA (L-JA), dark-Tween (D-TW) and dark-MeJA (D-JA). Values are the means of four individual chambers; error bars represent ±1 SE (ng h^−1^ g^−1^ DW). During the first 5 days after spraying MeJA, volatiles were measured in the light/dark consistent with the treatments (left panel). Between day 6 and day 10, trees grown in the light were measured again, and trees in the dark were measured after 1 h of re-exposure to light (right panel). Significant differences between treatments are indicated by different letters (*P* < 0.05).

Darkening significantly decreased emissions of all volatiles compared with trees grown in the light, with or without MeJA treatment (*P* < 0.05, HST or Wilcoxon’s rank-sum test; Figure 5). For trees grown in the light, spraying MeJA significantly increased emissions of monoterpene hydrocarbons (especially β-pinene and limonene) and linalool (Figure 5a and b), whereas sesquiterpenes remained relatively constant and MeSA significantly decreased (Figure 5c and d). For saplings exposed to 5 weeks of darkness, emissions of monoterpene hydrocarbons rapidly recovered after 1 h of re-exposure to light. They were also higher when saplings were sprayed with the mixture of Tween 20 and MeJA than with Tween 20 alone (Figure 5a). The two-way ANOVA showed that MeJA-induced monoterpene hydrocarbons did not vary with pre-treatments of light/dark (Darkness × MeJA interaction, *P* > 0.05), while MeJA-induced linalool emissions varied significantly (Darkness × MeJA interaction, *P* < 0.01; Table 2). Note that during re-illumination significant differences in emissions of monoterpene hydrocarbons were found independent of CO_2_ supply (see Figure S4 available as Supplementary Data at *Tree Physiology* Online). Unlike monoterpene hydrocarbons, emissions of linalool, sesquiterpenes and MeJA showed little change after re-exposure to light, independent of the MeJA application (Figure 5b–d).

## Discussion

Our study revealed trade-offs in allocation of carbon storage to growth and constitutive and induced SM, which are of critical importance for understanding and predicting tree defense under environmental stress. Under light limitation spruce saplings tend to decrease the mobilization of NSC stored in mature organs for biomass growth and production of constitutive SM in developing organs, and constitutive SM stored in mature organs cannot be remobilized and/or metabolized. Spraying Tween 20 had no effects on phenolic compounds and terpenoids across tissues, whereas spraying the mixture of Tween 20 and MeJA resulted in a reduction of stored NSC and a strong induction of flavan-3-ol and monoterpene synthesis only in the light. Emissions of monoterpene hydrocarbons may originate from stored resins, while emissions of linalool and sesquiterpenes were synthesized de novo and thus dependent on light and/or carbon availability.

### The NSC were preferentially used for constitutive biosynthesis of monoterpenes rather than of stilbenes and growth in developing organs, while SM stored in mature organs cannot be remobilized and recycled

We observed contrasting patterns for NSC and different types of SM in developing sink organs grown in the dark, where NSC and stilbenes significantly decreased much more than flavan-3-ols, while monoterpenes remained relatively constant. However, darkness treatment may trigger light signaling pathways and therefore did not allow partitioning the impacts of light and carbon availability. In a companion study where Norway spruce saplings were exposed to low CO_2_ conditions (Huang et al. 2019), NSC and phenolic compounds were also found to be more sensitive than monoterpenes. These results suggest that NSC are preferentially used for biosynthesis of monoterpenes rather than for phenolic compounds. Notably, concentrations of monoterpenes were less affected by darkness than by low CO_2_, possibly because low CO_2_ may increase stomatal conductance and thus led to a greater loss of monoterpenes through volatilization (Huang et al. 2018).

During darkness biomass growth of the developing sink organs was apparently fueled by NSC transported from the mature organs grown in previous years. Nevertheless, trees grown in the dark added much less biomass to developing organs compared with the light-grown control trees, while large amounts of NSC were still stored in mature needles and branches (>80 mg g^−1^) exposed to darkness. Similar results were found in other tree species exposed to shading (Piper and Fajardo 2016, Wiley et al. 2017, Weber et al. 2019), indicating that the mobilization of storage from source organs to growth of sink organs may have been downregulated in the dark. Our results are again in agreement with the results of Huang et al. (2019), which showed that more than 70% of soluble sugars were reserved in mature needles and branches under low CO_2_ while biomass growth was strongly limited (Huang et al. 2019). Our results highlight that low light/carbon availability, which can be induced by climate-driven disturbances (drought, heat, wildfire) and ecological interactions (e.g., competition), may reduce both mobilization and allocation of NSC storage to biomass growth and constitutive defense of developing sink organs, predisposing these organs to biotic attacks.

Given the lack of turnover of phenolic compounds (Huang et al. 2019) and terpenoids (Gershenzon et al. 2000), SM found in these organs were likely synthesized and stored prior to the darkness treatment. Hence, changes in SM in these mature organs under negative carbon balance may allow inferences on the accessibility of stored SM for remobilization and recycling under light and carbon limitation. Consistent with our hypothesis that SM are stored in specialized structures of mature organs that are not accessible, both concentrations of phenolic compounds and terpenoids remained relatively constant and were not dependent on NSC storage under complete darkness. Similarly, both phenolic compounds and monoterpenes stored in mature organs were not influenced by low CO_2_ conditions (Huang et al. 2019). These results suggest that stored SM could not be remobilized and/or metabolized in Norway spruce. While sequestering phenolic compounds and terpenoids appears to be without reward in the absence of herbivory, the sequestration allows avoidance of auto-toxicity that could result from the accumulation of constitutive SM.

### Light and/or carbon limitation constrained MeJA-induced production of SM by reducing local NSC availability in developing organs, but not in mature organs

Spraying MeJA strongly induced biosynthesis of flavan-3-ols and terpenoids in both developing and mature organs in the light, consistent with previous work on conifer species (Martin et al. 2002, Miller et al. 2005). However, the induction was much lower in mature organs than in developing organs. This suggests that constitutive levels of flavan-3-ols may have approached the maximum capacity of storage in mature organs before the application of MeJA. The different inducibility of flavan-3-ols between mature organs and developing organs in Norway spruce is consistent with the results from *Arabidopsis*, which showed that defense responses to biotic stress decline as organs develop (Berens et al. 2019) and plants mature (Mao et al. 2017).

**Figure 6.**
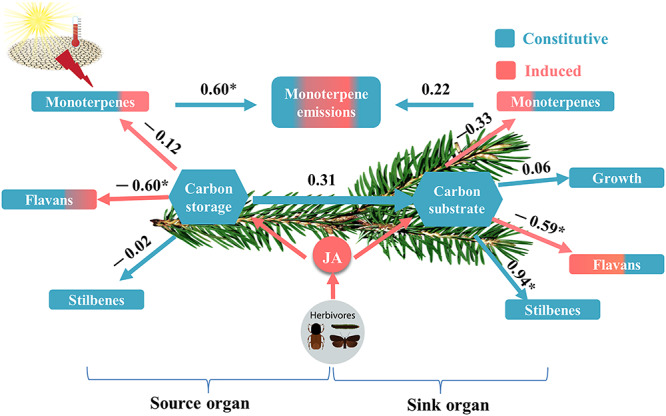
Relationships between NSC (sugars + starch) and SM (constitutively expressed plus induced) under situations of light limitation. NSC stored in previous-year, mature organs (source) and current-year, developing organs (sink) are denoted as carbon storage and carbon substrate, respectively. Values above arrows are standardized regression coefficients, which indicate the relative importance and direction of the relationships. Positive relationships are indicated with positive numbers and negative relationships with negative numbers. Asterisk indicates that the correlation is significant (*P* < 0.05) JA, jasmonate.

The induced biosynthesis of SM was tightly linked to a depletion of starch and soluble sugars (Figure 6), particularly sucrose, supporting the theory that herbivory can induce catabolism of NSC storage to support SM biosynthesis, as was found in pine trees (López-Goldar et al. 2016, Raffa et al. 2017, Roth et al. 2018). The mechanistic link between NSC and induced SM in the light may also explain why biosynthesis of flavan-3-ols and monoterpenes was not induced by MeJA in developing needles and branches in the dark, respectively. Concentrations of soluble sugars decreased to *c.* 40% (13 mg g^−1^) before spraying MeJA, which is close to the minimum threshold for survival (c. 5–10 mg g^−1^; Weber et al. 2018, Huang et al. 2019). Interestingly, although inducibility of SM was likely constrained by low NSC availability in developing needles and branches, large amounts of NSC in mature needles and branches (>50 mg g^−1^) were still available at the time of spraying MeJA. Regression analysis showed that the production of flavan-3-ols in developing organs was significantly correlated with the depletion of NSC, but not with changes in NSC stored in mature organs (Figure 6). This indicates that inducibility of SM in developing sink organs relies mainly on NSC that are stored locally rather than imported from mature, source organs. Our results corroborate a suggested physiological mechanism linking local sugar availability with bark beetle damage levels observed in field manipulations (Wiley et al. 2016).

Our results suggest that low light/carbon availability during environmental stress or from competition may limit the ability of trees to mobilize NSC for production of SM in response to biotic attacks. However, resources that are not transported to attacked or elicited leaves may be reserved to fuel regrowth after abiotic and biotic stress (Savage et al. 2016). We also observed that MeJA triggered the senescence of developing needles and branches grown in the dark, indicating that these saplings may have abandoned developing organs, possibly to ensure survival of mature organs and post-stress recovery.

### Light and carbon limitation altered the blend of constitutive and induced volatiles that may be important in tree defense against biotic agents

We observed different responses for different volatiles. Regardless of MeJA application, saplings emitted much less volatiles in the dark, in contrast to the results of Huang et al. (2018) which showed that emissions of volatiles increased under low CO_2_ conditions. The contrasting response of volatiles to darkness and low CO_2_ suggests that emissions of volatiles are highly dependent on light availability rather than carbon availability. Emissions of monoterpene hydrocarbons rapidly recovered after only 1 h of re-exposure to light. Re-illumination is known to cause stomata to open and thus may have allowed passive evaporation of monoterpene hydrocarbons stored in oleoresins. This explanation is supported not only by the significant positive correlations between their emissions and storage in mature organs but also by the similarities in the profiles. Interestingly, after re-illumination emissions of monoterpene hydrocarbons were also strongly induced by MeJA, while the tissue pool was not affected. Re-illumination activates photosynthetic electron transport and produces ATP and NADPH, the energy input required for de novo production of isoprenoid emissions (Niinemets et al. 2002*b*, Harrison et al. 2013). Hence, the induced emissions of monoterpene hydrocarbons may result from de novo synthesis.

Unlike monoterpene hydrocarbons, constitutive and induced emissions of linalool and sesquiterpenes remained low after re-illumination, with or without CO_2_ supply. We found no substantial storage of linalool or sesquiterpenes, indicating that their emissions are mainly synthesized de novo (Martin et al. 2003, Miller et al. 2005). Previous studies have shown that emissions of linalool were dependent on light (Niinemets et al. 2002*a*, Harley et al. 2014), inducible by MeJA (Martin et al. 2003, Miller et al. 2005), and were upregulated under low CO_2_ (50 p.p.m., Huang et al. 2018). In this study, however, constitutive and induced emissions of linalool failed to recover quickly after re-illumination, possibly because their biosynthesis may have been downregulated due to complete lack of carbon, ATP and NADPH following extended darkness.

Our results may provide a hypothetical link between environmental changes and tree interactions with insects. For example, direct defense via emissions of repellent and toxic monoterpene hydrocarbons may largely rely on stored compounds that were synthesized prior to severe stress. Emissions of linalool, sesquiterpenes and MeSA have been reported to act like airborne signals that can attract herbivore enemies or trigger defense in unattacked organs (McCormick et al. 2012), but their de novo synthesis can be suppressed by light and/or carbon availability. Our results thus highlight that environmental stress (severe drought, shade, defoliation) that changes light and/or carbon availability alters the blend of volatiles that may play an important role in tree defense against biotic agents.

## Conclusion and outlook

Trees exposed to abiotic stress (drought, fire and shading) are thought to be more susceptible to biotic stress. We conclude that low light and/or carbon availability may reduce mobilization and allocation of NSC storage to growth and constitutive and induced defense in spruce saplings. However, it should be noted that spruce saplings exposed to low carbon supply also rely on NSC storage for respiration, osmoregulation and transport (Huang et al. 2019), which can determine the availability of carbon for trade-offs between growth and defense. Comprehensive assessments of all these carbon fluxes (also in roots) would thus help to understand the role of carbon storage and partitioning in determining tree response to abiotic and biotic stress. Given the ontogenetic differences in carbon allocation, we propose that such assessments should be conducted on mature trees in the field.

## Supplementary Material

Supplemental_material_tpaa040

## References

[ref1] Berens ML, Wolinska KW, Spaepen S et al. (2019) Balancing trade-offs between biotic and abiotic stress responses through leaf age-dependent variation in stress hormone cross-talk. Proc Natl Acad Sci USA 116:2364–2373. 30674663 10.1073/pnas.1817233116 PMC6369802

[ref2] Biedermann PHW, Muller J, Gregoire JC et al. (2019) Bark beetle population dynamics in the Anthropocene: challenges and solutions. Trends Ecol Evol 34:914–924. 31262532 10.1016/j.tree.2019.06.002

[ref3] Dietze MC, Sala A, Carbone MS, Czimczik CI, Mantooth JA, Richardson AD, Vargas R (2014) Nonstructural carbon in woody plants. Annu Rev Plant Biol 65:667–687. 24274032 10.1146/annurev-arplant-050213-040054

[ref4] Erbilgin N, Krokene P, Christiansen E, Zeneli G, Gershenzon J (2006) Exogenous application of methyl jasmonate elicits defenses in Norway spruce (*Picea abies*) and reduces host colonization by the bark beetle *Ips typographus*. Oecologia 148:426–436. 16514534 10.1007/s00442-006-0394-3

[ref5] Franceschi VR, Krokene P, Christiansen E, Krekling T (2005) Anatomical and chemical defenses of conifer bark against bark beetles and other pests. New Phytol 167:353–376. 15998390 10.1111/j.1469-8137.2005.01436.x

[ref6] Galmán A, Petry WK, Abdala-Roberts L, Butrón A, Fuente M de la, Francisco M, Kergunteuil A, Rasmann S, Moreira X (2018) Inducibility of chemical defences in young oak trees is stronger in species with high elevational ranges. Tree Physiol 39:606–614. 10.1093/treephys/tpy139 30597091

[ref7] Gershenzon J, McConkey ME, Croteau RB (2000) Regulation of monoterpene accumulation in leaves of peppermint. Plant Physiol 122:205–214. 10631264 10.1104/pp.122.1.205 PMC58859

[ref8] Ghirardo A, Koch K, Taipale R, Zimmer I, Schnitzler JP, Rinne J (2010) Determination of *de novo* and pool emissions of terpenes from four common boreal/alpine trees by ^13^CO_2_ labelling and PTR-MS analysis. Plant Cell Environ 33:781–792. 20040067 10.1111/j.1365-3040.2009.02104.x

[ref9] Goodsman DW, Grosklos G, Aukema BH, Whitehouse C, Bleiker KP, McDowell NG, Middleton RS, Xu C (2018) The effect of warmer winters on the demography of an outbreak insect is hidden by intraspecific competition. Glob Chang Biol 24:3620–3628. 29808947 10.1111/gcb.14284

[ref10] Harley P, Eller A, Guenther A, Monson RK (2014) Observations and models of emissions of volatile terpenoid compounds from needles of ponderosa pine trees growing in situ: control by light, temperature and stomatal conductance. Oecologia 176:35–55. 25015120 10.1007/s00442-014-3008-5

[ref11] Harrison SP, Morfopoulos C, Dani KGS et al. (2013) Volatile isoprenoid emissions from plastid to planet. New Phytol 197:49–57. 23145556 10.1111/nph.12021

[ref12] Hartmann H, Trumbore S (2016) Understanding the roles of nonstructural carbohydrates in forest trees – from what we can measure to what we want to know. New Phytol 211:386–403. 27061438 10.1111/nph.13955

[ref13] Herms DA, Mattson WJ (1992) The dilemma of plants: to grow or defend. Q Rev Biol 67:283–335.

[ref14] Holopainen JK, Virjamo V, Ghimire RP, Blande JD, Julkunen-Tiitto R, Kivimäenpää M (2018) Climate change effects on secondary compounds of forest trees in the northern hemisphere. Front Plant Sci 9:1445. 30333846 10.3389/fpls.2018.01445 PMC6176061

[ref15] Howe GA (2004) Jasmonates as signals in the wound response. J Plant Growth Regul 23:223–237.

[ref16] Huang J, Hammerbacher A, Forkelová L, Hartmann H (2017) Release of resource constraints allows greater carbon allocation to secondary metabolites and storage in winter wheat. Plant Cell Environ 40:672–685. 28010041 10.1111/pce.12885

[ref18] Huang J, Hartmann H, Hellén H et al. (2018) New perspectives on CO_2_, temperature and light effects on BVOC emissions using online measurements by PTR-MS and cavity ring-down spectroscopy. Environ Sci Technol 52:13811–13823. 30335995 10.1021/acs.est.8b01435

[ref17] Huang J, Hammerbacher A, Weinhold A et al. (2019) Eyes on the future—evidence for trade-offs between growth, storage and defense in Norway spruce. New Phytol 222:144–158. 30289558 10.1111/nph.15522

[ref19] Huang J, Kautz M, Trowbridge AM et al. (2020) Tree defence and bark beetles in a drying world: carbon partitioning, functioning and modelling. New Phytol 225:26–36. 31494935 10.1111/nph.16173

[ref20] Hudgins JW, Christiansen E, Franceschi VR (2003) Methyl jasmonate induces changes mimicking anatomical defenses in diverse members of the Pinaceae. Tree Physiol 23:361–371. 12642238 10.1093/treephys/23.6.361

[ref21] Klein T, Hartmann H (2018) Climate change drives tree mortality. Science 362:758–758. 10.1126/science.aav6508 30442795

[ref22] Landhausser SM, Chow PS, Dickman LT et al. (2018) Standardized protocols and procedures can precisely and accurately quantify non-structural carbohydrates. Tree Physiol 38:1764–1778. 30376128 10.1093/treephys/tpy118 PMC6301340

[ref23] López-Goldar X, Sampedro L, Zas R (2016) Carbon starvation by light deprivation does not constrain the ability of young pines to produce induced chemical defences in response to a bark-chewing herbivore. Environ Exp Bot 130:141–150.

[ref24] Loreto F, Schnitzler JP (2010) Abiotic stresses and induced BVOCs. Trends Plant Sci 15:154–166. 20133178 10.1016/j.tplants.2009.12.006

[ref25] Mao Y-B, Liu Y-Q, Chen D-Y, Chen F-Y, Fang X, Hong G-J, Wang L-J, Wang J-W, Chen X-Y (2017) Jasmonate response decay and defense metabolite accumulation contributes to age-regulated dynamics of plant insect resistance. Nat Commun 8:13925. 28067238 10.1038/ncomms13925 PMC5233801

[ref26] Martin D, Tholl D, Gershenzon J, Bohlmann J (2002) Methyl jasmonate induces traumatic resin ducts, terpenoid resin biosynthesis, and terpenoid accumulation in developing xylem of Norway spruce stems. Plant Physiol 129:1003–1018. 12114556 10.1104/pp.011001 PMC166496

[ref27] Martin DM, Gershenzon J, Bohlmann J (2003) Induction of volatile terpene biosynthesis and diurnal emission by methyl jasmonate in foliage of Norway spruce. Plant Physiol 132:1586–1599. 12857838 10.1104/pp.103.021196 PMC167096

[ref28] Martínez-Vilalta J, Sala A, Asensio D, Galiano L, Hoch G, Palacio S, Piper FI, Lloret F (2016) Dynamics of non-structural carbohydrates in terrestrial plants: a global synthesis. Ecol Monogr 86:495–516.

[ref29] McCormick AC, Unsicker SB, Gershenzon J (2012) The specificity of herbivore-induced plant volatiles in attracting herbivore enemies. Trends Plant Sci 17:303–310. 22503606 10.1016/j.tplants.2012.03.012

[ref30] McDowell NG, Beerling DJ, Breshears DD, Fisher RA, Raffa KF, Stitt M (2011) The interdependence of mechanisms underlying climate-driven vegetation mortality. Trends Ecol Evol 26:523–532. 21802765 10.1016/j.tree.2011.06.003

[ref31] Miller B, Madilao LL, Ralph S, Bohlmann J (2005) Insect-induced conifer defense. White pine weevil and methyl jasmonate induce traumatic resinosis, de novo formed volatile emissions, and accumulation of terpenoid synthase and putative octadecanoid pathway transcripts in Sitka spruce. Plant Physiol 137:369–382. 15618433 10.1104/pp.104.050187 PMC548866

[ref32] Moreira X, Mooney KA, Rasmann S, Petry WK, Carrillo-Gavilan A, Zas R, Sampedro L (2014) Trade-offs between constitutive and induced defences drive geographical and climatic clines in pine chemical defences. Ecol Lett 17:537–546. 24818235 10.1111/ele.12253

[ref33] Najar A, Landhausser SM, Whitehill JGA, Bonello P, Erbilgin N (2014) Reserves accumulated in non-photosynthetic organs during the previous growing season drive plant defenses and growth in aspen in the subsequent growing season. J Chem Ecol 40:21–30. 24363094 10.1007/s10886-013-0374-0

[ref34] Newton A (2007) Aphid outbreaks. Nat Rep Clim Change 1:3.

[ref35] Niinemets Ü, Reichstein M (2003) Controls on the emission of plant volatiles through stomata: Differential sensitivity of emission rates to stomatal closure explained. J Geophys Res Atmos 108:4208.

[ref36] Niinemets Ü, Reichstein M, Staudt M, Seufert G, Tenhunen JD (2002 *a*) Stomatal constraints may affect emission of oxygenated monoterpenoids from the foliage of *Pinus pinea*. Plant Physiol 130:1371–1385. 12428002 10.1104/pp.009670 PMC166656

[ref37] Niinemets Ü, Seufert G, Steinbrecher R, Tenhunen JD (2002 *b*) A model coupling foliar monoterpene emissions to leaf photosynthetic characteristics in Mediterranean evergreen *Quercus species*. New Phytol 153:257–275.

[ref38] Pellissier L, Moreira X, Danner H, Serrano M, Salamin N, Dam NM, Rasmann S (2016) The simultaneous inducibility of phytochemicals related to plant direct and indirect defences against herbivores is stronger at low elevation. J Ecol 104:1116–1125.

[ref39] Piper FI, Fajardo A (2016) Carbon dynamics of *Acer pseudoplatanus* seedlings under drought and complete darkness. Tree Physiol 36:1400–1408. 27539732 10.1093/treephys/tpw063

[ref40] R Development Core Team (2016) R: a language and environment for statistical computing. R Foundation for Statical Computing, Vienna, Austria http://www.r-project.org.

[ref41] Raessler M, Wissuwa B, Breul A, Unger W, Grimm T (2010) Chromatographic analysis of major non-structural carbohydrates in several wood species—an analytical approach for higher accuracy of data. Anal Methods 2:532–538.

[ref42] Raffa KF (2014) Terpenes tell different tales at different scales: glimpses into the chemical ecology of conifer—bark beetle—microbial interactions. J Chem Ecol 40:1–20. 24337719 10.1007/s10886-013-0368-y

[ref43] Raffa KF, Mason CJ, Bonello P, Cook S, Erbilgin N, Keefover-Ring K, Klutsch JG, Villari C, Townsend PA (2017) Defence syndromes in lodgepole—whitebark pine ecosystems relate to degree of historical exposure to mountain pine beetles. Plant Cell Environ 40:1791–1806. 28543133 10.1111/pce.12985

[ref44] Roth M, Hussain A, Cale JA, Erbilgin N (2018) Successful colonization of lodgepole pine trees by mountain pine beetle increased monoterpene production and exhausted carbohydrate reserves. J Chem Ecol 44:209–214. 29302834 10.1007/s10886-017-0922-0

[ref45] Savage JA, Clearwater MJ, Haines DF, Klein T, Mencuccini M, Sevanto S, Turgeon R, Zhang C (2016) Allocation, stress tolerance and carbon transport in plants: how does phloem physiology affect plant ecology? Plant Cell Environ 39:709–725. 26147312 10.1111/pce.12602

[ref46] Scanlon JT, Willis DE (1985) Calculation of flame ionization detector relative response factors using the effective carbon number concept. J Chromatogr Sci 23:333–340.

[ref47] Schiebe C, Hammerbacher A, Birgersson G, Witzell J, Brodelius PE, Gershenzon J, Hansson BS, Krokene P, Schlyter F (2012) Inducibility of chemical defenses in Norway spruce bark is correlated with unsuccessful mass attacks by the spruce bark beetle. Oecologia 170:183–198. 22422313 10.1007/s00442-012-2298-8

[ref48] Seidl R, Muller J, Hothorn T, Bassler C, Heurich M, Kautz M (2016) Small beetle, large-scale drivers: how regional and landscape factors affect outbreaks of the European spruce bark beetle. J Appl Ecol 53:530–540. 10.1111/1365-2664.12540 PMC4816203 27041769

[ref49] Seidl R, Thom D, Kautz M et al. (2017) Forest disturbances under climate change. Nat Clim Chang 7:395–402. 28861124 10.1038/nclimate3303 PMC5572641

[ref50] Sevanto S, Dickman LT (2015) Where does the carbon go?—plant carbon allocation under climate change. Tree Physiol 35:581–584. 26109074 10.1093/treephys/tpv059

[ref51] Sevanto S, McDowell NG, Dickman LT, Pangle R, Pockman WT (2014) How do trees die? A test of the hydraulic failure and carbon starvation hypotheses. Plant Cell Environ 37:153–161. 23730972 10.1111/pce.12141 PMC4280888

[ref52] Smith AM, Stitt M (2007) Coordination of carbon supply and plant growth. Plant Cell Environ 30:1126–1149. 17661751 10.1111/j.1365-3040.2007.01708.x

[ref53] Dam NM van, De Jong TJ, Iwasa Y, Kubo T (1996) Optimal distribution of defences: are plants smart investors? Funct Ecol 10:128–136.

[ref55] Weber R, Schwendener A, Schmid S, Lambert S, Wiley E, Landhäusser SM, Hartmann H, Hoch G (2018) Living on next to nothing: tree seedlings can survive weeks with very low carbohydrate concentrations. New Phytol 218:107–118. 29424009 10.1111/nph.14987

[ref54] Weber R, Gessler A, Hoch G (2019) High carbon storage in carbon-limited trees. New Phytol 222:171–182. 30451299 10.1111/nph.15599

[ref57] Wiley E, Rogers BJ, Hodgkinson R, Landhäusser SM (2016) Nonstructural carbohydrate dynamics of lodgepole pine dying from mountain pine beetle attack. New Phytol 209:550–562. 26256444 10.1111/nph.13603

[ref56] Wiley E, Hoch G, Landhäusser SM (2017) Dying piece by piece: carbohydrate dynamics in aspen (*Populus tremuloides*) seedlings under severe carbon stress. J Exp Bot 68:5221–5232. 29036658 10.1093/jxb/erx342 PMC5853906

[ref58] Wobbrock J.O., Findlater L., Gergle D., Higgins J.J. 2011 The aligned rank transform for nonparametric factorial analyses using only anova procedures Proceedings of the SIGCHI Conference on Human Factors in Computing Systems. ACM, Vancouver, BC, Canada, pp 143–146.

[ref59] Züst T, Agrawal AA (2017) Trade-offs between plant growth and defense against insect herbivory: an emerging mechanistic synthesis. Annu Rev Plant Biol 68:513–534. 28142282 10.1146/annurev-arplant-042916-040856

