# Supplementary material for: Production of constitutive and induced secondary metabolites is coordinated with growth and storage in Norway spruce saplings
Source: Tree Physiol. 2020 Apr 9;40(7):928–42. doi: 10.1093/treephys/tpaa040 (PMC7325531; doi:10.1093/treephys/tpaa040)
Supplement: Supplemental_material_tpaa040 [file supplemental_material_tpaa040.pdf]

Supplemental information

Production of constitutive and induced secondary metabolites is coordinated with growth and storage in Norway spruce saplings

Authors: Jianbei Huang, Alexander Rücker, Axel Schmidt, Gerd Gleixner, Jonathan Gershenzon, Susan Trumbore, and Henrik Hartmann

The following supplemental material is available for this article:

Figure S1 Chromatograms of monoterpenes measured by GC-FID.

Figure S2 Concentrations of total soluble sugars, starch and total NSC (sugars + starch).

Figure S3 Concentrations of flavan−3−ols, stilbenes, and monoterpenes.

Figure S4 Emissions of monoterpene hydrocarbons after re-exposure to light with or without CO2 supply.

Figure S5 Concentrations of jasmonic and salicylic acids.

Table S1 Two-way ANOVA results on concentrations of individual compounds.

Table S2 Concentrations of glucose, sucrose and fructose

Figure S1 Chromatograms of monoterpenes measured by GC-FID.

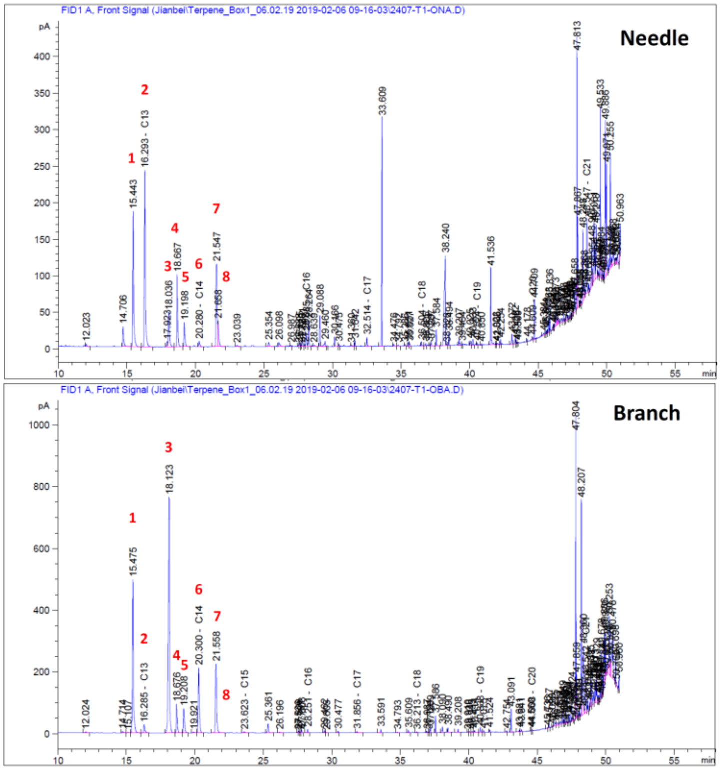

Compounds

1. α-Pinene
2. Camphene
3. β-Pinene
4. 1,9-Decadiene
5. Mycrene
6. 3-Carene
7. Limonene
8. 1, 8-Cineole

Figure S2 Concentrations of soluble sugars (a-d), starch (e-h), and total NSC (soluble sugars + starch, i-l) in developing and mature needles and branches of *Picea abies* grown under Light-MeJA (squares, yellow-red line), Dark-Tween (triangles, dark line), Dark-MeJA (triangles, dark-red line), compared to control, Light-Tween (squares, yellow line). The red lines indicate MeJA treatment under either light or darkness. Error bars indicate coefficients of variation and propagated standard errors. Significant treatment effects were calculated based on the raw concentrations and are indicated by filled symbols (P<0.05).

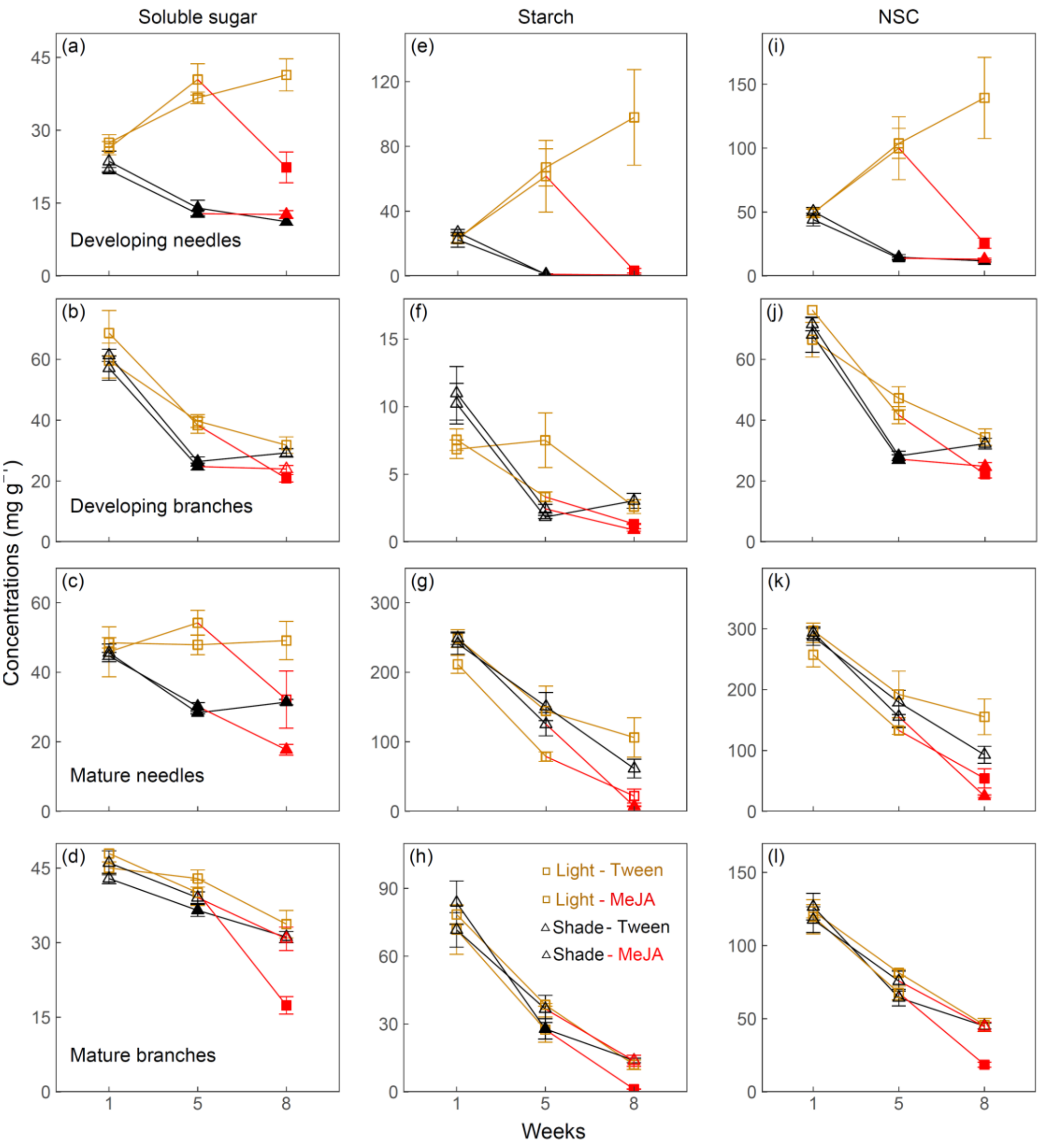

Figure S3 Concentrations of flavan−3−ols (a-d), stilbenes (e-h), and monoterpenes (i-l) in developing and mature needles and branches of *Picea abies* grown under Light-MeJA (squares, yellow-red line), Dark-Tween (triangles, dark line), Dark-MeJA (triangles, dark-red line), compared to control, Light-Tween (squares, yellow line). The red lines indicate MeJA treatment under either light or darkness. Error bars indicate coefficients of variation and propagated standard errors. Significant treatment effects were calculated based on the raw concentrations and are indicated by filled symbols (P<0.05).

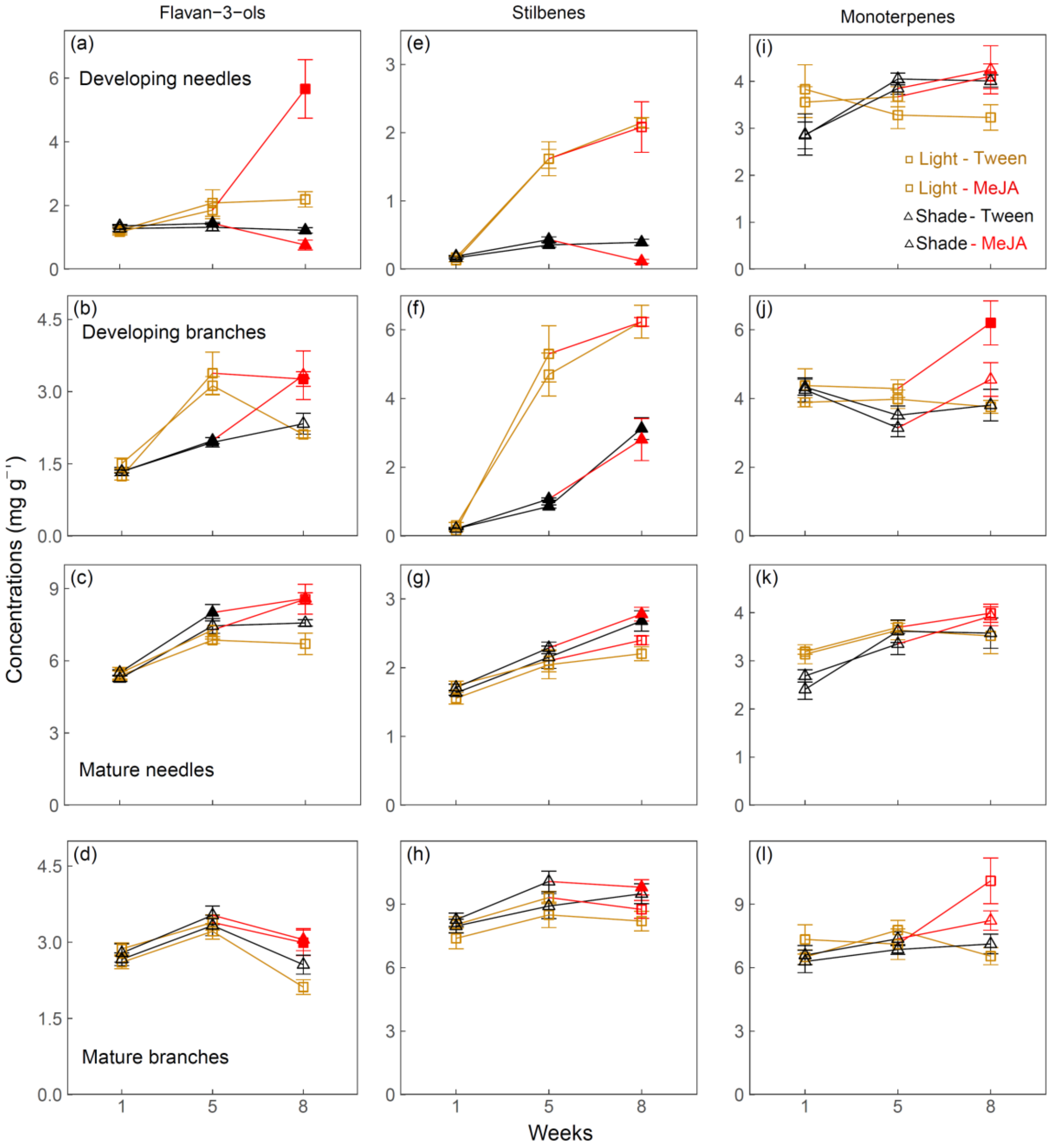

Figure S4 Emissions (ng h^{-1} g^{-1} DW) of monoterpene hydrocarbons from the canopy of *Picea abies* grown either in light or dark, followed by spraying methyl jasmonate (MeJA) dissolved in Tween 20 or Tween 20 only: Light-Tween (L-TW), Light-MeJA (L-JA), Dark-Tween (D-TW), Dark-MeJA (D-JA). Volatiles from light treatments were measured at either 400 or 0 ppm CO_2; volatiles from dark treatments were measured one hour after re-illumination with either 0 or 400 ppm CO_2.

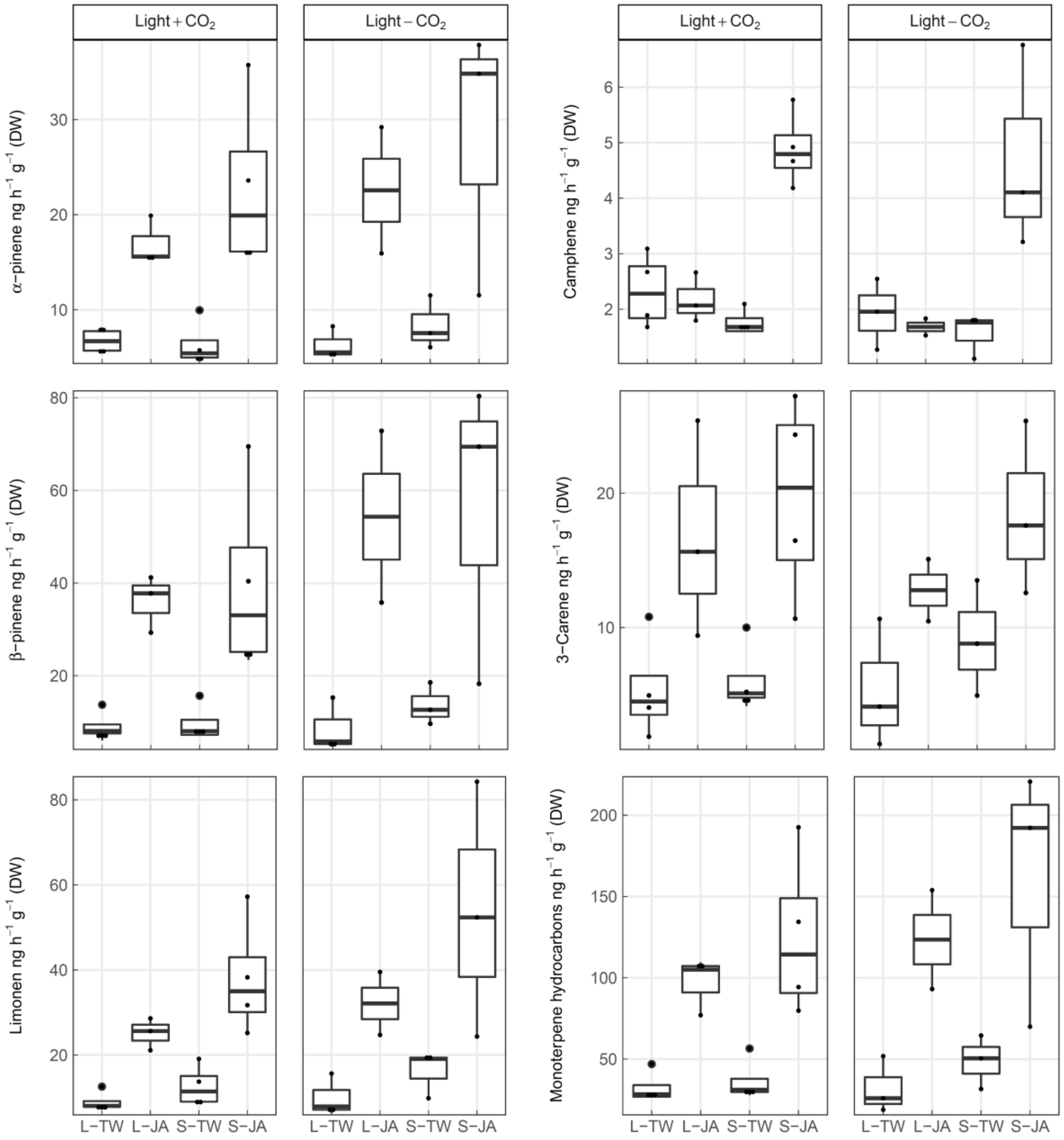

Table S1 Effects of darkness and methyl jasmonate (MeJA) and their interactions on the concentrations of individual soluble sugars, flavan-3-ols, stilbenes, and monoterpenes in developing needles and branches of *Picea abies* at week 8.

	Developing needles			Developing branches		
Pools	Dark	MeJA	Dark×MeJA	Dark	MeJA	Dark×MeJA
Glucose	0.92	<0.01	0.70	0.08	0.30	0.58
Sucrose	<0.01	<0.01	<0.01	0.41	<0.01	0.12
Fructose	0.26	0.83	0.04	0.08	0.03	0.36
Catechin	<0.01	0.04	<0.01	0.21	<0.01	0.27
Gallocatechin	0.01	0.01	0.01	<0.01	<0.01	<0.01
Proanthocyanidin	<0.01	0.08	0.02	0.11	0.05	0.20
Astringin	<0.01	0.64	0.25	<0.01	0.29	0.05
Isorhapontin	<0.01	0.12	0.95	<0.01	0.86	0.34
α -pinene	0.30	0.26	0.58	0.06	<0.01	0.04
β-pinene	0.36	0.13	0.41	0.06	<0.01	0.03
Camphene	0.15	0.21	0.58	0.25	0.35	0.51
Myrcene	0.55	0.19	0.23	0.03	<0.01	0.04
3−carene	0.17	0.01	0.85	0.78	0.06	0.40
Limonene	0.40	0.21	0.46	0.19	<0.01	0.06

P values were determined using two-way ANOVA.

Table S2 Concentrations of glucose, sucrose and fructose in developing and mature needles and branches of *Picea abies* grown under Light-Tween, Light-MeJA Dark-Tween, Dark-MeJA. Values are mean (SE) of four individual saplings.

	Developing needles			Developing branches			Mature needles			Mature branches		
	Glucose	Sucrose	Fructose	Glucose	Sucrose	Fructose	Glucose	Sucrose	Fructose	Glucose	Sucrose	Fructose
Light-Tween	7.3 (3.6)	30.3 (4.6)	3.7 (3.0)	8.6 (0.6)	21.8 (2.9)	1.3 (0.1)	19.1 (3.9)	19.9 (3.2)	10.0 (2.6)	5.8 (0.2)	26.1 (2.1)	1.9 (0.5)
Light-MeJA	14.4 (2.2)	2.2 (0.6)	5.8 (1.2)	9.4 (0.6)	10.4 (1.8)	1.2 (0.2)	21.4 (6.2)	2.1 (0.3)	8.7 (2.1)	4.5 (0.6)	12.7 (1.1)	0.2 (0.2)
Dark-Tween	4.9 (0.2)	3.3 (0.4)	3.0 (0.3)	7.8 (0.4)	20.3 (1.1)	1.2 (0.1)	14.6 (0.6)	7.4 (1.1)	9.4 (0.6)	8.2 (0.2)	19.7 (0.6)	3.1 (0.5)
Dark-MeJA	12.7 (0.8)	0.0 (0.0)	0.0 (0.0)	7.9 (0.5)	15.2 (1.4)	0.8 (0.1)	12.8 (1.0)	0.2 (0.2)	4.7 (0.5)	7.8 (0.3)	20.2 (1.5)	2.8 (0.7)
